# The Significance of Equipment Availability and Anesthesia Educational Conferences to Decision-Making for EKG Lead V5 Abnormalities

**DOI:** 10.7759/cureus.53620

**Published:** 2024-02-05

**Authors:** Kimberly L Skidmore, Joseph Drinkard, Henson M Randall, Giustino Varrassi, Sahar Shekoohi, Alan D Kaye

**Affiliations:** 1 Department of Anesthesiology, Louisiana State University Health Sciences Center, Shreveport, USA; 2 Department of Medicine, Edward Via College of Osteopathic Medicine, Monroe, USA; 3 Department of Pain Medicine, Paolo Procacci Foundation, Rome, ITA

**Keywords:** postoperative myocardial infarction, troponin, preoperative evaluation, electrocardiogram monitoring, myocardial ischemia

## Abstract

Introduction

To predict postoperative myocardial infarction rates in patients who undergo noncardiac surgery, the Canadian Cardiovascular Society Guidelines on Perioperative Cardiac Risk Assessment and Management recommends assessment of brain natriuretic peptide (BNP) in certain patients. Serial troponins are measured if the BNP level is elevated. In certain cases, Revised Cardiac Risk Index (RCRI) alone does not perform well, for example, during vascular surgery. Cardiac events occur in 20% of all vascular surgery patients. The odds ratio for such events is 9.2 if ST segments were depressed by 1 mm intraoperatively (relative to the PR interval) within the first 48 hours postoperatively. Increasing the number of cables and pads from three to five for electrocardiogram (EKG) increases the sensitivity from around 30% to over 80% for ischemic events relative to a formal EKG stress test, and then the monitor continuously displays not only lead II but also lead V5.

Methods

Our hypothesis was that raising awareness about diagnostic and therapeutic options to reduce the risk of postoperative myocardial infarction would increase the use of five pads. We conducted open-ended surveys at six hospitals to assess the reasons for choosing three pads. In our university hospital practice, we measured a cross-sectional incidence of using three pads before and, once again, a month after an intervention during a single morning. Several resident conferences encouraged the use of five pads. Education included weekly lectures and informal discussions with other staff during surgery, demonstrating that using five pads allows interrogation of an entire 12-lead EKG. In comparison, three pads only allow viewing three leads.

Results

At baseline, only three pads were available in 96% of our 23 operating rooms. Five cables were available in eight of those surgeries, but two were taped off to the side. Surveys unveiled scarcity of equipment and, more importantly, disempowerment (i.e., knowing how to diagnose or when to treat ischemia). After several conferences, the prevalence of equipment availability of only three pads fell to 47%.

Conclusions

Education enumerated details of recognizing ischemic configurations of ST depression. Next, education revealed methods to interrupt the progression of ischemia to infarction such as elevated blood pressure and hematocrit, reducing heart rate, and calling a cardiology consultant if the anesthesiologist wishes to draw serial troponins. Barriers to implementing an enhanced recovery after surgery (ERAS) pathway began with a need for more access to manage stress tests or optimize blood pressure medications after a preoperative anesthesia evaluation. The intraoperative barrier was knowing what to do if ST depression occurs. Therefore, we began raising awareness by encouraging the addition of an element of a future ERAS pathway, adding a cost of only $1 to monitor lead V5. Future ERAS pathways can include preoperative stress tests and consults, as found in published guidelines.

## Introduction

We wished to measure the availability of equipment for five EKG cables (rather than three cables) perioperatively in the community because cardiovascular disease is becoming more common. We aimed to discover barriers to education about preoperative risks for ischemia, the sensitivity of lead V5 for diagnosis, and algorithms of therapies. In a landmark prospective investigation of 474 California veterans undergoing vascular surgery, 18% suffered cardiac events [1]. Myocardial infarction had an odds ratio of 9.2 if preceded by ST depressions. In comparison, vascular surgery itself incurred only an odds ratio of 1.8. Odds ratios are defined as the chance of an outcome in the presence or absence of a risk factor and are regarded as useful statistical tools for predicting events. Care was administered according to the usual standards, with only three pads being used intraoperatively, and cardiac enzymes were drawn only when clinically indicated. Most often, the symptom that causes clinicians to suspect ischemia is dyspnea. Clinicians were blinded to the Holter EKG data during this research protocol. Intraoperatively, approximately 25% of patients experienced ST depression 60 ms after the J point, at an average heart rate of 70 beats per minute. This compared favorably to around 41% of patients from arrival in the recovery room to 48 hours later, with concurrent heart rates above 90. EKG monitoring ended at 48 hours. Although most postoperative myocardial infarctions are a day or two after surgery, the early warning sign of ST depression (marking reversible ischemia) may cause anesthesiologists to begin therapies that circumvent the permanent damage of myocardial infarction.

Since the era two decades ago of that study, Americans appear to present for surgery with higher risks of obesity, diabetes, and adverse cardiovascular outcomes. As Americans continue to experience delayed primary care after the COVID-19 pandemic, worsening their cardiac conditions, it is incumbent upon anesthesiologists to increase vigilance regarding preoperative risk stratification and intraoperative goal-directed hemodynamics. Of inpatients requiring surgeries, approximately 1% die within 30 days, evenly divided between myocardial infarction and bleeding or infection [2]. In a study of millions of elective surgeries, ranging from gallbladder or colon to heart or lung, the 30-day mortality rate was 1.3% for Black men over age 65 [3]. This mortality rate of 1.3% was statistically higher than the mortality rate of approximately 0.85% for Black women, White men, and White women. Among several possible reasons, Black men experience a higher prevalence of poverty and lack of access to continuous primary care, enabling optimization of each comorbidity with medications and social support. In support of this theory, when only the subgroup undergoing emergency surgery was considered, much of the race-associated difference in mortality evaporated.

A notable top-down collaborative that began at Stanford demonstrated success in all California hospitals. After the collaborative group sent emissaries to 99 hospitals to share toolbox algorithms, they found that postpartum hemorrhage was reduced by 9% for Black women and by 2% for White women. Much of the improvement was attributed to shifting the culture away from overuse of cesarean section [4]. Another group standardized cesarean section perioperative care and found a significantly reduced length of hospital stay [5]. This was especially true for Black women. The California Maternal Quality Care Collaborative (CMQCC) turned recently to the most common cause of maternal mortality, cardiovascular disease, accounting for around one in three maternal deaths in California and the United States. Most of these deaths were preventable. In half of the mortality cases, cardiovascular etiologies were undiagnosed until these researchers reviewed charts postmortem [6]. Consequently, protocols must be implemented by disseminating toolkits, that is, educational algorithms, to preoperative clinics before effectively reducing maternal mortality from cardiovascular disease [7]. Those authors showed that one group centered on one university can obtain Title V federal funding. Their toolkits included triggers for drawing a brain natriuretic peptide (BNP), chest X-ray, EKG, stress test, hypertension medications, cardiology consults, and data defining barriers to implementing these protocols. As we shall discuss, grassroots work and obtaining buy-in from the bottom-up via informal communication among frontline clinicians must also occur [8]. Staff can become disenfranchised, feeling their actions do not matter [9]. Therefore, our study began with open-ended interviews of frontline staff, including anesthesia technicians, certified registered nurse anesthetists (CRNAs), student registered nurse anesthetists (SRNAs), anesthesia faculty, and residents [10]. We began with one simple, measurable step, adding available equipment and discussions regarding two additional EKG cables, in efforts to raise general awareness about reversible myocardial ischemia.

Enhanced recovery after surgery (ERAS) consists of multimodal pathways from preoperative evaluation throughout the hospital stay, designed to improve the quality of outcomes. One study proved that if ERAS compliance is not ensured within all aspects of the culture of safety, the average length of hospital stay is increased 3.5-fold [11]. Another study showed that at an 800-bed level-one trauma center, the hospital administration needed to address the teamwork of various clinicians to enhance a culture shift to reduce infections. Beginning from the bottom-up is essential, and thus favoring the design of quality improvement pathways, the administration hosted lunch parties, allowing anyone to submit a paper containing their suggestions for naming the campaign and the best small steps they believe would have the most significant impact. When the purchase of hand gel (volume in liters per month) doubled for the operating rooms, in a sudden but sustained manner, surgical site infections were cut in half. The hand gel cost only around half a million dollars but was predicted to save well over 17 million dollars annually [9].

After constructing any ERAS protocol, first, compliance data must be collected. Second, team leaders must interpret why clinicians are failing to comply. Finally, educational efforts need to target the problem areas. One hard outcome must be tied closely to one new behavior such as cutting surgical site infections temporally linked to a single variable (e.g., the hand gel) among a bundle of simple-to-implement yet high-impact therapies [9].

An ERAS protocol should begin preoperatively. One example that could be more widely implemented is a practice guideline for preoperative evaluation, updated recently in the Canadian Cardiovascular Society Guidelines on Perioperative Cardiac Risk Assessment and Management. Risk stratification in the preoperative days per these guidelines recommends assessment of BNP in certain patients (e.g., if over age 65 or age 45 if the Revised Cardiac Risk Index (RCRI) is above zero). If BNP is elevated, troponin is drawn daily [12]. Regarding predicting myocardial infarction, the RCRI alone is not considered very sensitive [13]. RCRI, however, is very helpful in risk stratification for predicting the duration of hospital stay and costs in general [14]. Intraoperative and postoperative troponin values are suggested where clinically indicated, guided by abnormal EKG, hypotension, or dyspnea, among other signs or symptoms. Logically, if serial troponins are being drawn, one should also continuously monitor an EKG with as high a sensitivity as feasible during the de facto “stress test” experienced during surgery, as many stress hormones continue to impact ischemia in the recovery room and beyond.

Nationally, whether three or five EKG pads are the community standard intraoperatively is unclear. It is agreed that patients who have a high chance of ST depression should have five pads. Aimed explicitly at reducing myocardial infarctions, the factors most closely associated with risk remain controversial [15]. A single lead, which is often lead II, is continuously displayed when three pads and cables are available. However, if clinicians are interested in one or two clicks to see advanced settings, three pads can display leads I, II, and III. Likewise, five pads are usually configured to continuously display leads II and V5. If clinicians wish to click “advanced” settings and “see all,” they can display seven leads out of a 12-lead EKG. If the pad labeled “V” is moved from right to left, from V1 through V6 positions, then all 12 leads of a full EKG can be obtained using five pads. Lead “V” allows additional V7, V8, and V9 for posterior ischemia or right-sided V leads in any configuration desired. A baseline photograph can be taken before stress-test situations such as trachea intubation, especially extubation, and in cases of pain with concomitant tachycardia postoperatively.

If ST depression conformations suggest ischemia, interventions such as careful titration of intravenous metoprolol can prevent mortality [16-18]. Other interventions might include elevating blood pressure or sending an image of all leads to a cardiologist. Researchers have found that the duration (even a few minutes) of mean arterial blood pressure below 60 mmHg or systolic below 100 mmHg correlated with a higher risk of both renal and myocardial dysfunction [19]. Next, anesthesiologists are responsible for transfusion to a hematocrit above 28% if cerebral or myocardial ischemia is suspected [20]. Clinical follow-up studies include frequent 12-lead EKG and troponin levels to guide a team of consultants regarding formal stress tests or, more immediately, interventions possible in the coronary angiography suite.

## Materials and methods

Our null hypothesis was that conferences educating 24 anesthesia residents about the various benefits of monitoring five EKG pads rather than three would not increase the incidence of using five pads. Our primary outcome measurement was the availability of equipment and the application of five pads. Our secondary outcome was listing barriers to the availability of equipment and formal education for what interpretation and therapies should follow abnormal EKG readings from five pads. Alongside this education, informal discussions with anesthesia technicians, faculty, CRNAs, and SRNAs were viewed to be equally important in collecting lists of barriers. After several years of school and passing the written board examination, an SRNA becomes a CRNA.

Before the educational conferences began, we surveyed the six surgical suites wherein our team had experience within the last year: an out-of-state academic operating room suite, a community hospital in town, an ambulatory surgery center nearby, an obstetrical suite, a community hospital in a neighboring state, and our 400-bed level-one trauma hospital. We measured the number of patients with three pads (with three cables) placed on their chests versus five pads (with five cables). After a whole month of resident education formal conferences to encourage compliance with our informal EKG-related five-pad ERAS pathway and informal communications with staff during surgeries, we remeasured our incidence of equipment availability of only three pads on a weekday morning, around 9 am again.

The first group we addressed was the anesthesia residents because our medical school and conferences lend themselves to intellectual curiosity and teamwork through altruism. The next group we addressed is SRNAs and their teachers, the CRNAs. The final group was the faculty of our anesthesiology department because they supervise others. Informal interviews (at random times and intensity, stemming from feasibility issues) with all the aforementioned types of staff and anesthesia technicians, who were historically instructed to tape two of five cables away, were conducted utilizing open-ended questions about why they were only monitoring three leads.

Educational efforts began with a faculty member’s grand rounds about what factors lead to cardiac failure as the leading cause of maternal mortality and how CMQCC discovered BNP and stress tests or echocardiograms preoperatively can help risk-stratify patients who display specific signs and magnitudes of tachypnea, tachycardia, or dyspnea [4]. The following week, a resident led a conference focused on how failing to discuss checklists in ICU handoff contributes to missing the etiology of hypotension. Resident core lectures by faculty included textbook images showing ST depression from ischemia versus those from electrolyte disturbances or normal variants, with a particular emphasis on lead V5. CRNAs, faculty, and anesthesia technicians were interviewed informally throughout the month, forming a simple generalized campaign to increase the use of V5. When two of the five cables were found to be taped to the side, these investigators untaped them and placed two more pads, discussing with anyone present the significant role of V5.

The main new intervention was one conference for residents, including a PowerPoint presentation led by a resident. He empowered all 24 residents to see the following three points. First, preoperatively, anesthesiologists can predict which patients need further workup. Second, intraoperatively, we should learn how to interpret the five-pad EKG, providing over 80% sensitivity, versus the usual three pads with around 30% sensitivity, relative to a treadmill stress test [21]. Third, perioperatively, and most critically important, the resident's lecture described simple steps anesthesiologists can take to reverse temporary ischemia, thus preventing a permanent heart attack [22]. The clinical impact of failure to diagnose ischemia to avoid a “heart attack” was emphasized in terms of commonly missed signs of ST depression.

The stress of surgery, especially as transitioning to the recovery phase, with surges of pain and catecholamines, is a missed opportunity to observe a “stress test” and intervene before infarction. The resident's PowerPoint presentation showed the Canadian preoperative evaluation guidelines, the location where ST segment depression of 1 mm is detected, and a table of possible treatments to prevent infarction (Table 1). Residents were educated on the GUSTO-1 trial of over 40,000 patients, which revealed that EKG lead changes in acute myocardial infarction, including inferior wall (II, III, aVF), anterior (V1-V4), apical (V5-V6), and lateral (aVL, I). Subgroup analysis showed that non-Q-wave infarctions resulted in half the mortality rate of Q-wave infarctions of just 1% [23]. Residents were reminded that perioperative myocardial infarctions, however, have much higher mortality rates. Intraoperative ischemia is usually supply and demand in nature and is more easily reversed than a fixed coronary stenosis. The EKG location of ST segment depression of 1 mm during such subendocardial ischemia is usually diffuse, present in several leads except in aVR, where it can be reciprocal elevation [24]. Thus, the culture of safety was encouraged through altruism, as it was found most helpful in increasing volunteering for blood donation [25]. The conference emphasized (as in the Canadian guidelines) that the measurement of BNP and troponin, where indicated, and calling consultants or delaying surgery, whether intraoperatively or preoperatively, are connected to improved survival.

**Table 1:** Educational toolkit presented at a resident-led conference for the entire Department of Anesthesia Examples of EKG images of artifacts or ischemia were shown. The literature was reviewed cited in this introduction. CVP, central venous pressure; LVH, left ventricular hypertrophy; PACU, post-anesthesia care unit; RBBB, right bundle branch block; TWI, T wave inversion

1) Predict ischemia if	Consider	Next consider
Revised Cardiac Risk Index > 0 or age > 65	Brain natriuretic peptide	Troponin
All intraoperative and PACU patients	Five cables and pads EKG creates a full 12-lead EKG.	1x save photograph. See all leads. Move V1-V6.
2) Interpret ST segment		
>1-mm depression 60 ms after QRS relative to PR interval.	If ST depression of more than 1 mm in any lead (except in aVR, where ST elevation is over 1 mm) persists beyond 30 minutes, consider steps to limit infarction. Ischemia less than 30 minutes is often reversible.	Consider pause surgery. Draw troponin. Call cardiologist.
ST elevation > 0.1 mV (=1 mm) or elevation > 0.2 mV in (=2 mm)	In two contiguous limb leads or two precordial leads	Injury evolving into transmural infarction
Old myocardial infarct	Q > 30 ms in leads: I, II, avL, avF, or V4-V6	Or any Q in V1-V3
Evolving myocardial infarct posteriorly	ST depression V2 or V3 (=reciprocal changes)	Add V7-V9 pads on back to detect ST elevation
Old myocardial infarct posteriorly	R waves in V1-V3	Tall T waves V1-V3
Artifacts: RBBB, LVH	V5 TWI may be ischemia	If ST > 1-mm depressed
LBBB can be ischemia if:	ST elevation: R height > 0.3 ST elevation > 1-mm ST elevation > 5-mm ST depression > 1 mm	ST > 1 mm if dominant R wave if dominant S wave in V1-V3
Pulmonary embolism	RBBB, S1Q3T3	=S in I, Q, and TWI in lead III
Pericarditis	PR depression or PR elevation	ST elevation in V6, II (and depressed in aVR)
Hypokalemia	V4	ST depression upsloping, late T
Hyperkalemia	Narrow	Tall T waves
Digitalis	V4	ST depression downsloping
Hypocalcemia	V4	Long QT, small T
Hypercalcemia	Short ST segment	ST upsloping elevation
Catecholamines	PR and ST segments	Depressed on same level
J-point elevation = normal variant	V6 ST elevation< 25% T wave	“Early repolarization” is ST elevation in: aVL, I, II, avF, V4-V6
Digitalis	Downsloping	ST segment depressed
3) Treatments	Target	Using
Raise perfusion and resistance	MAP > 70 mmHg	Phenylephrine
Dilate coronary arteries	Nitroglycerin	Or nicardipine
Raise hematocrit	Over 28%	Raise tissue Svo_2_ or cerebral oximetry
Raise cardiac output	Flo-Trak	Fractional shortening
Optimize preload	CVP	Arterial line monitor pulse pressure variation
4) Follow-up care	BNP, troponin	12-lead EKG, ultrasound. Call cardiologist.

## Results

The comments derived from our three investigators during a month of dozens of open-ended interviews were categorized as four types of problems post hoc. (1) The clinicians believed that adding lead V5 information would not change their diagnosis of any medical problems. (2) They also believed that none of the therapies such as BNP, troponin, beta blocker, blood transfusion, 12-lead EKG, or cardiologist consults would be able to predict or prevent infarction. (3) They did not have a formal verbal handoff checklist that included cardiac issues. (4) There is a scarcity of staff and supplies, including EKG pads or five-cable EKG wires, and availability of consultants by known phone numbers. They mentioned that while other hospitals nearby sometimes use five pads but sometimes use three, the reasons are inconsistent. Once in the ICU, new cables are applied with five new disposable pink cables for each single-patient use. Five permanent wires are available in some recovery room beds and only three in some.

Regarding the prevalence of three versus five leads, Table 2 shows various cross-sectional survey results from different practice settings our investigators contacted. At one out-of-state university hospital, 100% of intraoperative and recovery room beds have a five-pad system. At one in-state community outpatient ambulatory surgery center and another inpatient obstetrical suite, 100% of rooms have only three cables. At an in-state community hospital, 100% of intraoperative rooms have five cables, but the recovery room monitors only have three cables; however, after a shift in the purchase of disposable pink EKG cables that always have five cables, the recovery room continued use of all five pads. An out-of-state community hospital in the South used three cables only when, as determined by an anesthesiologist, there was a low risk of perioperative ischemia.

**Table 2:** Cross-sectional survey results from different practice settings where our investigators have worked recently Some permanent grey cables have only three cables. Also, some permanent grey cables have five cables, but the anesthesia technician was instructed to tape away two of them to discourage use. Disposable pink cables could be cleaned and reused but always contain five cables. Number (N) is approximate given that some locations are non-operating rooms.

Practice setting location for assessment of the availability of the number of EKG cables	Three cables available / approximately N operating rooms
Our university baseline	96% / 23
Our university after a month of conferences	47% / 23
Our obstetrical suite	100% / 2
Other university	0% / 52
Ambulatory center	100% / 3
Other community hospital	90% / 8
Other community hospital	80% / 15

In November of 2023, we noted that all EKG cables were reusable and could be moved to a transport monitor in our hospital, causing three operating rooms to be left without a main monitor module receptacle, probably during transport to ICU, without replacing the original main receptacle. In our 23 operating rooms, only three pads were available for use in 96% of rooms with anesthetized patients. Five cables were available in eight operating rooms, but two were tied away. In four rooms, five cables were available, but there was no patient present. After a month of conferences about preoperative Canadian guidelines, the prevalence of three pads available fell to 47%, where eight rooms had five cables available. Still, in two rooms, two cables were taped away and unavailable, and three rooms had only three cables available.

One CRNA mentioned that after observing hypotension, he noted ST depression, and it was only because of our campaign that he decided to draw a troponin and call a cardiologist. The surgery was stopped in the middle of the procedure, and the patient had a stress test showing an old and fixed defect. A resident in the recovery room, for a different patient, noted ST depression and decided to draw a troponin and call the hospitalist. They ordered a stress test the next day. A faculty member diagnosed ST depression in the recovery room, on yet another patient, obtained an entire 12-lead EKG, administered intravenous metoprolol, and obtained another 12-lead EKG that had less but some ST depressions. The cardiologist was consulted and, immediately, a coronary stent was placed.

## Discussion

Identifying barriers to the implementation of the ERAS pathway

Surveying clinical partners is the first step toward identifying why a new ERAS protocol is failing. Common reasons include lack of teamwork in a challenging work environment, lack of self-directed small-group management, no previous history of ERAS protocols, and high turnover or burnout of staff. Teamwork must be present, showing mutual respect, or the orchestra may not perform what the conductor is directing [8]. Other stakeholders might sideline anesthesiologists, yet they can insist on and achieve higher quality care through informal communications [10].

Leadership providing positive reinforcement for each educational barrier

Once the barriers are identified, one leader must discover a concrete method to encourage compliance regarding each barrier. Buy-in from clinical partners will be required in team meetings. Finally, leadership must reassess a simple hard outcome variable closely linked to the intervention. For example, surveys of handoff in intensive care units reveal that a lack of formalized checklists and uniform team communication worsens outcomes and team building [26].

The biggest strength of our study is the proof that grassroots informal communication increases one intraoperative (availability of V5 data) small step, likely tied to increased vigilance for diagnosis of ischemia. We add to the literature the baseline incidence and settings where three pads are chosen over five pads. We present concise educational tables and a discussion for other groups to apply to triage ST segment changes throughout the recovery room duration. Logically, this increased awareness of rising cardiovascular disease should encourage five pads in more anesthesia settings.

A drawback to our study is that only one time point was measured, without regard to maintaining and enforcing this education at regular intervals. Another weakness of our study is that our hospital system has a small size yet a rapid turnover of new students. Generalizing these results across operating rooms nationally is also difficult because intraoperative ischemia is less common than postoperative ischemia. The additional cost of two more pads could be recouped by reusing or recycling cables rather than purchasing single-use cables; continuing the five-pad EKG throughout the recovery room stay should be financially neutral. The largest hurdle is the applicability to all practice settings of five pads, while many clinicians do not appreciate the implications of various abnormal results.

Many anesthesia clinicians are not aware that the option exists to create a 12-lead EKG in the operating room. A baseline image can be photographed. Obtaining more perioperative studies (troponin or BNP) or cardiology consults in a clinic led by someone reinforcing the latest major practice guidelines creates a de facto ERAS quality improvement pathway. The anesthesiologist’s preoperative evaluation should include an RCRI. Consider a baseline BNP, troponin, and a point-of-care transthoracic ultrasound if the patient desaturates or is unable to walk up two flights of stairs and is scheduled for a high-stress surgery [2]. Through this pathway, anesthesiologists will know when to consult cardiologists for stress tests or improved diabetes, cholesterol, and blood pressure titration of medications. When already undergoing surgery, a point-of-care ultrasound can often clarify (body habitus, notwithstanding) the etiology when it is unexplained [27, 28]. In over half of patients with unexplained hypotension, transesophageal echocardiography will reveal a new diagnosis and lessen mortality rates [29].

**Figure 1:**
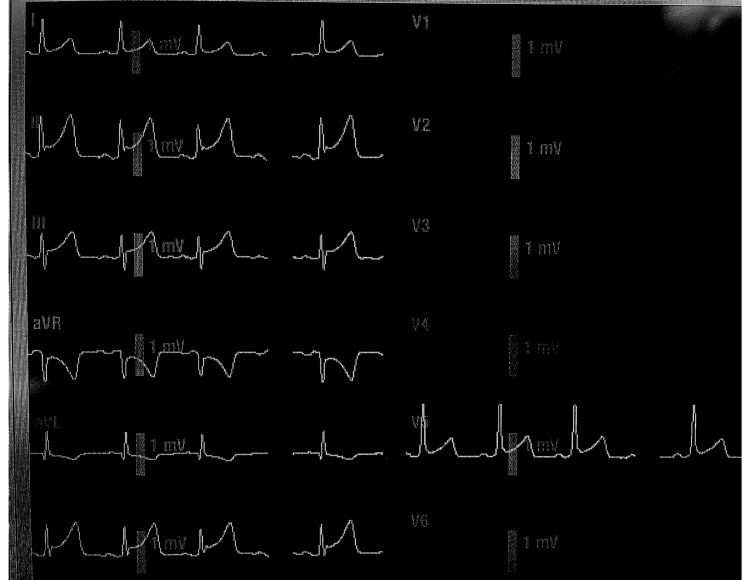
Normal variant ST elevation Photograph taken from a General Electric standard anesthesia monitor by the first author of this article in our operating room in 2022. In this J-point elevation case, note the ST elevation in II, III, aVF, and V5. This variant can be associated with some minor concurrent diseases.

The American Heart Association recently recommended that all high-risk patients have preoperative and a postoperative troponin levels measured if the results would change clinical decisions [2]. Most perioperative ischemia presents within 24 hours of surgical incision and is supply and demand in nature [1]; 20% of inpatients show troponin leak, yielding a hazard ratio of 2.28 for 30-day mortality [2]. A clinically relevant rise of 20% in troponin would justify interventions such as beta-blockers, coronary angiography, or consultations [15]. Our study increased the availability of equipment and ease of use for allowing increased sensitivity for detecting intraoperative ST segment depression and, therefore, awareness should be raised for drawing an intraoperative troponin and calling for consultations, where appropriate. The left bundle branch block is an example of a condition that reduces the sensitivity of ST depression monitoring. Yet, baseline images and some criteria are still helpful for early warning of ischemia [30]. Anesthesiologists must be familiar with another common normal variant artifact called “J-point elevation” (Figure 1).

**Figure 2:**
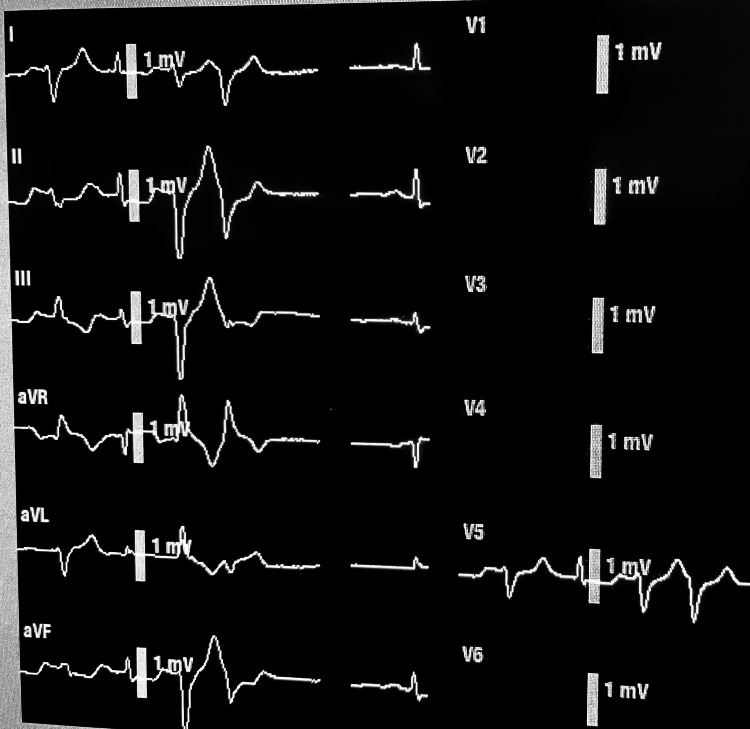
ST depression ST depression of more than 1 mm compared to the PR segment is significant. Also, T wave inversion is often present in ischemia. Photograph taken from a General Electric standard anesthesia monitor by the first author of this article in our operating room in 2022. The first, third, and fourth QRS complexes are wide and polymorphic, which is also sometimes indicative of ischemia. Electrolyte imbalance (e.g., pH, potassium, calcium, magnesium, and phosphorus) should be ruled out. After the second QRS, which appears similar to the baseline configuration in as much as having a normal QRS duration and axis, new ST depression of >1 mm between 60 and 80 ms (i.e., 2 mm) after the J point is present (compared to this PR interval baseline) and therefore suggestive of subendocardial ischemia. If the duration continues longer than around 30 minutes, without correction of supply and demand imbalance, permanent myocardial infarction may result. The bright vertical line in the middle of the second QRS represents the scale bar of 10-mm height, where each 1 mm represents 0.1 mV. Many machines report the name of one of the 12 leads and the precise millimeters of ST depression or elevation. Grids can be superimposed for 1-mm markings. Some machines allow the user to move the PR interval and the ST depression locations manually with respect to time on the horizontal axis. Some machines automatically impose the timing and display numerical ST results. Pacemaker-provided impulses may be added and appear as small white vertical lines before the usual green color of the EKG tracing.

Anesthesiologists should continue to monitor intraoperative ischemia with more sensitive instruments. Five pads cost an additional $1, but logically, it would be cost-effective when diffuse ST depression greater than 1 mm emerges (Figure 2). The weakness of our study is that it is small and bottom-up, yet only if all clinicians feel mutual respect and are individually empowered to determine feasible small steps that dramatically improve patient care will they comply with overriding ERAS protocols. Future efforts will be to incorporate this additional EKG V5 into a comprehensive ERAS pathway, beginning with uniform education of residents in the preoperative clinic. Vazirani et al. investigated a before and after retrospective study at a university-associated Veterans Affairs preoperative clinic [31]. Before the intervention, anesthesiology residents ran the clinic, often changing rotations and individuals. After the intervention, only faculty hospitalists with one set of nurse practitioners ran the clinic. The nurse practitioners were taught to follow algorithms from a few printed publications about practice guidelines. These nurse practitioners were empowered to write prescriptions for beta blockers, stress tests, insulin, and smoking cessation support groups. Significantly more stress tests and beta blockers were ordered in the new mode, and many last-minute cancellations and fatal heart attacks were prevented.

## Conclusions

In the present investigation, we measured the use of equipment in operating rooms before and after grand rounds and informal discussions related to the clinical importance of the availability of five-lead EKG cables. To conserve cable systems, methods to clean and reuse these cables were formulated as well. We surveyed and reported the prevalence of three-lead cable systems at similar institutions in order to provide a context for comparison of common clinical standards. In summary, ongoing education as to the significant role of five-lead EKG cables in clinical anesthesia practice improved understanding and quality of care for trainees and staff.
